# A novel multi-feature fusion attention neural network for the recognition of epileptic EEG signals

**DOI:** 10.3389/fncom.2024.1393122

**Published:** 2024-06-19

**Authors:** Congshan Sun, Cong Xu, Hongwei Li, Hongjian Bo, Lin Ma, Haifeng Li

**Affiliations:** ^1^Faculty of Computing, Harbin Institute of Technology, Harbin, China; ^2^Shenzhen Academy of Aerospace Technology, Shenzhen, China

**Keywords:** epilepsy, EEG, Hilbert spectrum, grayscale recurrence plot, self-attention mechanism

## Abstract

Epilepsy is a common chronic brain disorder. Detecting epilepsy by observing electroencephalography (EEG) is the main method neurologists use, but this method is time-consuming. EEG signals are non-stationary, nonlinear, and often highly noisy, so it remains challenging to recognize epileptic EEG signals more accurately and automatically. This paper proposes a novel classification system of epileptic EEG signals for single-channel EEG based on the attention network that integrates time-frequency and nonlinear dynamic features. The proposed system has three novel modules. The first module constructs the Hilbert spectrum (HS) with high time-frequency resolution into a two-channel parallel convolutional network. The time-frequency features are fully extracted by complementing the high-dimensional features of the two branches. The second module constructs a grayscale recurrence plot (GRP) that contains more nonlinear dynamic features than traditional RP, fed into the residual-connected convolution module for effective learning of nonlinear dynamic features. The third module is the feature fusion module based on a self-attention mechanism to assign optimal weights to different types of features and further enhance the information extraction capability of the system. Therefore, the system is named HG-SANet. The results of several classification tasks on the Bonn EEG database and the Bern-Barcelona EEG database show that the HG-SANet can effectively capture the contribution degree of the extracted features from different domains, significantly enhance the expression ability of the model, and improve the accuracy of the recognition of epileptic EEG signals. The HG-SANet can improve the diagnosis and treatment efficiency of epilepsy and has broad application prospects in the fields of brain disease diagnosis.

## 1 Introduction

Epilepsy is a kind of brain disease caused by the abnormal hypersynchronous firing of neurons in the brain, which poses a great threat to the life and health of patients (Acharya et al., 2013). Therefore, an accurate epilepsy diagnosis is of great clinical significance in reducing the harm caused by epileptic seizures to patients. Electroencephalography (EEG) is the most commonly used and effective procedure for diagnosing epilepsy (Noachtar and Rémi, 2009). The diagnosis of epilepsy is a continuous and long-term process (Sazgar and Young, 2019; Jang and Lee, 2020). Moreover, the characteristic pattern of epileptic seizures varies greatly among different patients and even within the same patient (Ren et al., 2023). Therefore, the diagnosis of epilepsy and the pattern analysis of epileptic seizures are usually carried out by neurologists through the detailed analysis of a large number of EEG data by visual detection and manual annotation (Peng et al., 2022). Since EEG signals are nonlinear, non-stationary, highly noisy, and tend to be of long duration, manual judgment to analyze EEG signals is very time-consuming and subject to the subjective judgment of the clinician (Andrzejak et al., 2001; San-Segundo et al., 2019; Hamavar and Asl, 2021). Therefore, more efficient automated detection and analysis methods have received much attention recently. This work will explore automatic and accurate recognition techniques of epileptic EEG signals to assist neurologists in analyzing EEG signals, reduce the burden of neurologists, and improve the efficiency of epilepsy diagnosis and treatment.

For the classification methods of epileptic EEG signals, scholars mainly use statistical analysis-based methods, traditional machine learning and deep learning methods. Gao et al. (2018) propose a statistical analysis-based method to detect seizures. First, they compute joint time-domain features and use the auto-regressive (AR) linear model to model the data. Then, based on the non-parametric statistical test of random power martingale (RPM), the decision is made. Das et al. (2018) extracted time-domain and frequency-domain features of EEG signals based on variational mode decomposition (VMD) and then detected epileptic seizure events by thresholding. Chen et al. (2019) used various distance measurement methods, such as Bhattacharyya distance, to solve the feature similarity of the power spectrum features based on short-time Fourier transform (STFT) of EEG signals at different moments and then detected the EEG signals by null hypothesis test. The above method has the advantages of easy implementation and fast detection speed. Since EEG signals are non-stationary signals, they are easily disturbed by noise generated by brain activity, and the extracted features are easily statistically unstable, leading to inaccurate detection results. In addition, scholars have conducted a lot of research on the classification of epileptic EEG signals based on machine learning and deep learning. Wang et al. (2017) extracted time-domain, frequency-domain, and time-frequency-domain features of EEG signals based on wavelet transform (WT), extracted nonlinear features based on information theory, and then combined the two types of features for epileptic seizure detection by machine learning methods such as k-nearest neighbor classification (KNN) and support vector machine (SVM). Lu et al. (2021) extracted several nonlinear features, such as sample entropy and Higuchi’s fractal dimension, and combined them with SVM for epileptic EEG classification. Then, they found that phase space reconstruction and Poincaré section can improve the recognition accuracy of epileptic EEG signals. Jang and Lee (2020) use the wavelet transform (WT) and phase space reconstruction (PSR) to extract features and then input features to the neural network with weighted fuzzy membership (NEWFM) to detect seizure. Sui et al. (2021) proposed a time-frequency hybrid network (TFHybridNet) based on STFT and a convolutional neural network (CNN) for epileptic focus localization. Varlı and Yılmaz (2023) propose a combined deep learning model based on CNN and long short-term memory (LSTM) to detect seizures. This model uses continuous wavelet transform (CWT) and STFT methods to input the signal conversion time-frequency image to the CNN module and the raw EEG signal to the LSTM module. Compared with traditional machine learning models and statistical analysis-based methods, deep learning models have stronger learning ability and better performance. Current deep learning methods mainly focus on the construction of deep network structures. Combining the non-stationary and nonlinear inherent signal characteristics of EEG with deep learning technology to improve detection accuracy needs further research.

Empirical mode decomposition (EMD) is a non-stationary signal analysis method widely used in the study of epileptic EEG recognition (Mahjoub et al., 2020; Lu et al., 2023). EMD decomposes EEG signals into several linear combinations of intrinsic mode functions (IMF). However, due to the mode mixing problem in EMD, false components in the obtained IMF will adversely affect the EEG analysis. In our previous work, we proposed an improved EMD method named adaptively optimized masking empirical mode decomposition (AOMEMD) (Sun et al., 2024). AOMEMD can effectively alleviate the mode mixing problem of EMD so that the obtained IMFs can effectively capture the underlying physics of EEG. By applying the Hilbert transform (HT) to the IMFs, the Hilbert spectrum (HS) of the EEG can be constructed for high-resolution time-frequency representation of EEG signals. Compared with STFT and CWT methods, this method does not need to set the basis function in advance and has high adaptability and flexibility. Therefore, in this paper, time-frequency features of EEG are represented based on AOMEMD and HT.

The recurrence plot (RP) is a nonlinear time series analysis method that can reveal hidden dynamic characteristics in EEG signals in the form of images (Eckmann et al., 1987; Huang et al., 2023). The traditional RP is a binary symmetric square matrix, usually using the recurrence quantification analysis (RQA) method to extract the structural features of RP for classification recognition. Since the traditional RP cannot reflect detailed time series information, scholars have proposed various improved RP methods. Hatami et al. (2017) skipped the threshold segmentation step in the process of RP construction and combined the gray-level texture image of RP with CNN to classify the time series. Khosla et al. (2022) proposed an un-thresholded recurrence plot (URP) and used the fractal weighted local binary pattern (URP-FWLBP) method to extract the texture features to classify epileptic seizure types. Experiments show that the URP-FWLBP method is better than the traditional method based on RQA. Considering the nonlinear, dynamic, and complex EEG signal, this paper combines the time-frequency feature based on HT with the nonlinear and non-stationary features based on RP to classify epileptic EEG signals.

Therefore, in this paper, we propose a novel system combining nonlinear dynamic features of EEG and time-frequency features extracted by non-stationary time-frequency analysis methods with deep learning techniques to classify epileptic EEG signals automatically. The proposed system is based on a self-attention mechanism to fuse time-frequency features of the HS and nonlinear dynamic features of the grayscale recurrence plot (GRP) to detect epileptic EEG signals for single-channel EEG. So, we call the proposed system HG-SANet. Several classification tasks on the Bonn EEG database and the Bern-Barcelona EEG database verify the performance of the proposed system for the classification of epileptic EEG signals.

## 2 Materials and methods

In this section, the public dataset used in this paper is first introduced. Secondly, the proposed approach of seizure detection in EEG signals is elaborated. Finally, the experimental setup of this paper is introduced.

### 2.1 Dataset and data pre-processing

**FIGURE 1:**
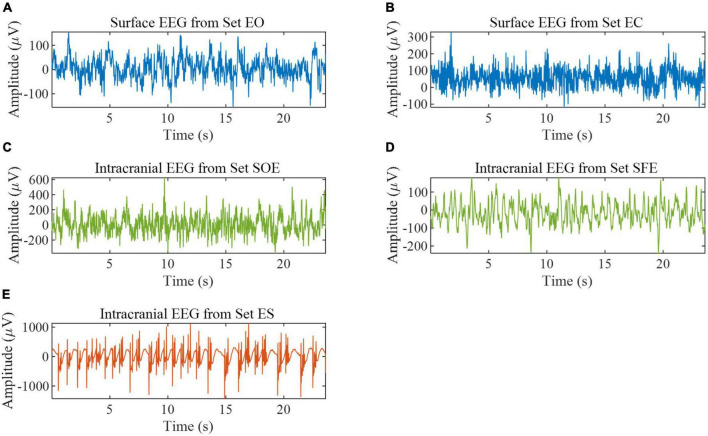
EEG samples from the Bonn EEG database. **(A)** Example of set EO. **(B)** Example of set EC. **(C)** Example of set SOE. **(D)** Example of set SFE. **(E)** Example of set ES.

In this paper, two datasets are used. The first dataset is the Bonn EEG time series (Andrzejak et al., 2001). The dataset consists of five sets (denoted A, B, C, D, and E in the original reference) of single-channel EEG segments from healthy volunteers and epilepsy patients, with a signal sampling frequency of 173.61 Hz and a duration of 23.6 s per sample. In order to better distinguish the five subsets, the names of the five subsets are changed to A (denoted EO), B (denoted EC), C (denoted SOE), D (denoted SFE), and E (denoted ES). Each set has 100 recordings and is described in Table 1. Some samples are shown in Figure 1. All EEG signals are digitally band-pass filtered over a range of 0.53∼40 Hz. We used all the samples in this database for experiments to verify the effectiveness of the proposed method in epilepsy detection. We split the data to expand the size of the dataset (Varlı and Yılmaz, 2023). The data is divided into a segment of 512 sample points; the distance between segments is 128 sample points, the last one sample points of the data are deleted, and the final data is divided into 29 segments.

**TABLE 1:** The details of five sets in the Bonn EEG time series.

Set	New name	Subjects	Conditions	Electrodes
A	EO	Healthy volunteers	Eyes open	Surface
B	EC	Healthy volunteers	Eyes closed	Surface
C	SOE	Epilepsy patients	Seizure-free interval from outside the epileptogenic zone	Intracranial
D	SFE	Epilepsy patients	Seizure-free interval from epileptogenic zone	Intracranial
E	ES	Epilepsy patients	Epileptic seizure	Intracranial

**FIGURE 2:**
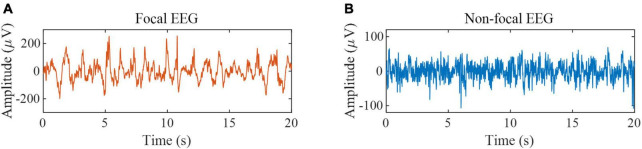
EEG samples from the Bern-Barcelona EEG database. **(A)** Example of focal EEG signals. **(B)** Example of non-focal EEG signals.

The second dataset is the Bern-Barcelona EEG database (Schindler et al., 2012). The dataset consists of focal and non-focal EEG segments during seizure-free periods from five epilepsy patients, with a signal sampling frequency of 1,024 Hz and a duration of 20 s per sample. Each class has 3,750 samples. If the channel is in the epileptogenic region, its label is focal; otherwise, its label is non-focal. The database is preprocessed as follows: (1) Samples are down-sampled to 512 Hz; (2) All EEG signals are digitally band-pass filtered over a range of 0.5∼150 Hz using a fourth-order Butterworth filter and phase distortions are minimized using forward filtering and backward filtering (Schindler et al., 2012). We used all the samples in this database for experiments to verify the effectiveness of the proposed method in epileptic focus localization. Some samples are shown in Figure 2. According to the previous works (Fasil and Rajesh, 2019), the data is divided into a non-overlapping segment of 1,024 sample points to expand the size of the dataset, and the final data is divided into 10 segments.

All the EEG signals in two datasets are normalized by the following Equation 1 to keep all data at the same scale, helping to improve recognition performance.


(1) $$\tilde{\text{x}}=\frac{\text{x}-\mu }{\sigma }$$

where ***x*** is the input signal, *μ* is the mean of the signal, and *σ* is the standard deviation of the signal.

### 2.2 The proposed framework

**FIGURE 3:**
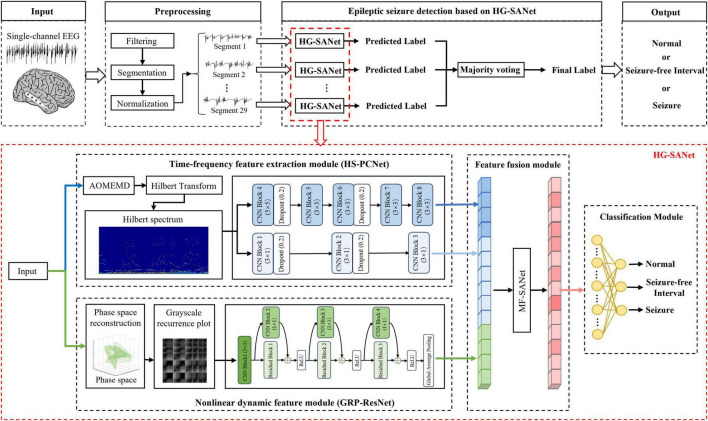
The overview of the proposed epileptic seizure detection system. The cortical model in the figure is from the literature (Andrzejak et al., 2001).

The overview of the system based on the proposed HG-SANet is shown in Figure 3. The HG-SANet consists of three modules: EEG time-frequency feature extraction module based on HS and two-channel parallel convolutional neural network (HS-PCNet), nonlinear dynamic feature extraction module based on GRP and residual networks (GRP-ResNet), and multi-domain feature fusion module based on self-attention mechanism (MF-SANet). Below, we first introduce the construction method of HS and GRP and then introduce the network structure of each module.

#### 2.2.1 AOMEMD-based Hilbert spectrum

In this part, we use AOMEMD and HT to construct Hilbert spectrum. For a single-channel EEG signal *x*(*t*), the AOMEMD is first used to decompose *x*(*t*) into a finite number of IMFs and a residue. Therefore, *x*(*t*) can be represented as Equation 2:


(2) $$x\,\left(t\right)=\sum_{k=1}^{n_{i\,m\,f}}c_{k}\,\left(t\right)+r\,\left(t\right)$$

where *c*_*k*_(*t*) (*k* = 1, 2,…, *n*_*imf*_) is the *k*th IMF and *r*(*t*) represents the residue. The frequency of the *n*_*imf*_ IMFs decreases from the first to the *n*_*imf*_th in order. In this work, we use the AOMEMD without the optimization strategy, which can save computation time while maintaining performance (Sun et al., 2024). The AOMEMD obtains IMFs through the following sifting process and the details of EMD are referred to the work of Huang et al. (1998).

Step 1: Input the signal *x*(*t*). Initialize *k* = 1 and *r*_*k*–1_(*t*) = *x*(*t*). The number of phases is *n*_*p*_.

Step 2: Determine the amplitude ${\bar{a}}_{k}$ and frequency ${\bar{f}}_{k}$ of the *k*th group masking signal *v*_*k*_(*t*) with resulted IMFs by applying EMD to *r*_*k*–1_(*t*).

Step 3: Construct the *k*th group masking signal $v_{k\,j}\,\left(t\right)={\bar{a}}_{k}\,c\,o\,s\,\left[2\,\pi \,{\bar{f}}_{k}\,t+2\,\pi \,\left(j-1\right)/n_{p}\right]$, (*j* = 1, 2,…,*n*_*p*_). Obtain the *k*th IMF $c_{k}\,\left(t\right)=\left[\sum_{j=1}^{n_{p}}{\text{EMD}}_{1}\,\left(r_{k-1}\,\left(t\right)+v_{k\,j}\,\left(t\right)\right)\right]/n_{p}$, where EMD_1_ (⋅) represents to obtain the first IMF using EMD.

Step 4: Update *r*_*k*_(*t*) = *r*_*k*–1_(*t*)- *c*_*k*_(*t*) and *k* = *k*+1. If *r*_*k*–1_(*t*) fulfils termination criterion, *r*(*t*) = *r_*k*–_*_1_(*t*); otherwise, go to step 2 and execute the loop.

For the obtained *c*_*k*_(*t*) (*k* = 1, 2,…, *n*_*imf*_) by AOMEMD, we use the HT to obtain the instantaneous frequency *f*_*k*_(*t*) and instantaneous amplitude *a*_*k*_(*t*) of *c*_*k*_(*t*). The formula for *y*_*k*_(*t*) obtained by applying the HT to *c*_*k*_(*t*) is shown in Equation 3 (Huang et al., 1998):


(3) $$y_{k}\,\left(t\right)=\frac{1}{\pi }\,\text{p}.\text{v}.\int_{-\infty }^{+\infty }\frac{c_{k}\,\left(\tau \right)}{\tau -t}\,d\tau$$

where p.v. is the cauchy principal value. Then, *f*_*k*_(*t*) and *a*_*k*_(*t*) are solved as shown in Equations 4, 5:


(4) $$f_{k}\,\left(t\right)=\frac{1}{2\,\pi }\cdot \frac{d}{d\,t}\,\left(\arctan\,\frac{y_{k}\,\left(t\right)}{c_{k}\,\left(t\right)}\right)$$

(5) $$a_{k}\,\left(t\right)=\sqrt{c_{k}^{2}\,\left(t\right)+y_{k}^{2}\,\left(t\right)}$$

Then the amplitude distribution of *x*(*t*) with frequency and time is the Hilbert spectrum (HS), denoted as *HS*(*f*, *t*), expressed as follows Equation 6:


(6) H⁢S⁢(f,t)=Re⁢(∑k=1ni⁢m⁢fak⁢(t)⁢ei⁢∫-∞t2⁢π⁢fk⁢(τ)⁢dτ)

**FIGURE 4:**
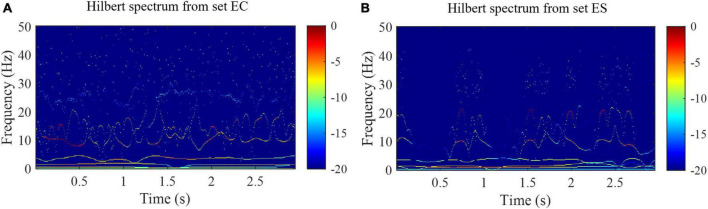
Hilbert spectra from the set EC and set ES of the Bonn EEG database. **(A)** Hilbert spectrum from the set EC. **(B)** Hilbert spectrum from the set ES.

Where Re represents the real part and i is the imaginary unit. *HS*(*f*, *t*) is a two-dimensional matrix with a time resolution equal to the sampling period (Molla and Hirose, 2007). Examples of HS are shown in Figure 4. As shown in Figure 4, the time-frequency distribution of samples from set EC and set ES is quite different.

#### 2.2.2 Grayscale recurrence plot

**FIGURE 5:**
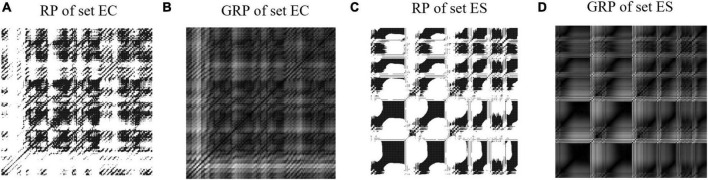
Examples of recurrence plots and grayscale recurrence plots from the set EC and set ES. **(A)** Recurrence plot from set EC. **(B)** Grayscale recurrence plot from set EC. **(C)** Recurrence plot from set ES. **(D)** Grayscale recurrence plot from set ES.

For a single-channel EEG signal *x*(*t*) of length *T*_*EEG*_, the RP is computed as the following. First, according to Takens’ embedding theory (Takens, 1985), a phase space is reconstructed for *x*(*t*), and a phase point in this space is denoted as *s*_*n*_ and *n* = 1, 2, …, *T*_*EEG*_ – *T*_*ps*_ (*m*–1), where *T*_*ps*_ is the time delay and *m* is the embedding dimension. *T*_*ps*_ and m can be selected using mutual information (MI) and false nearest neighbor (FNN) methods, respectively (He et al., 2023). Second, the RP is defined according to Equation 7 below:


(7) $$R\,P\,\left(n,j\right)=\left\{ \begin{array}{c} 1,\varepsilon \geq ||s_{n}-s_{j}||\\ 0,\varepsilon <||s_{n}-s_{j}|| \end{array}\right.,$$

$$n,j=1,2,\ldots ,T_{E\,E\,G}-T_{p\,s}\,\left(m-1\right)$$

where ε is the distance threshold and || ⋅ || is the Euclidean norm. By assigning a black dot to the RP element (*n*, *j*) of *RP*(*n*, *j*) = 1 and a white dot to the RP element (*n*, *j*) of RP(*n*, *j*) = 0, a binary square image of an RP can be obtained, as shown in Figures 5A,C.

Binary square images constructed using the threshold method lose a lot of information, so we convert the RP to a grayscale intensity image (named grayscale RP, GRP). The examples of GRP are shown in Figures 5B,D. The GRP is defined according to Equation 8 below (Chen and Shi, 2019):


(8) $$G\,R\,P\,\left(n,j\right)=\frac{||s_{n}-s_{j}||-\min\,\left(||s_{n}-s_{j}||\right)}{\max\left(||s_{n}-s_{j}||\right)-\min\,\left(||s_{n}-s_{j}||\right)},$$

$$n,j=1,2,\ldots ,T_{E\,E\,G}-T_{p\,s}\,\left(m-1\right)$$

#### 2.2.3 Network structure of the HG-SANet

In this section, the network structure in each module of the HG-SANet is described in detail.

**FIGURE 6:**
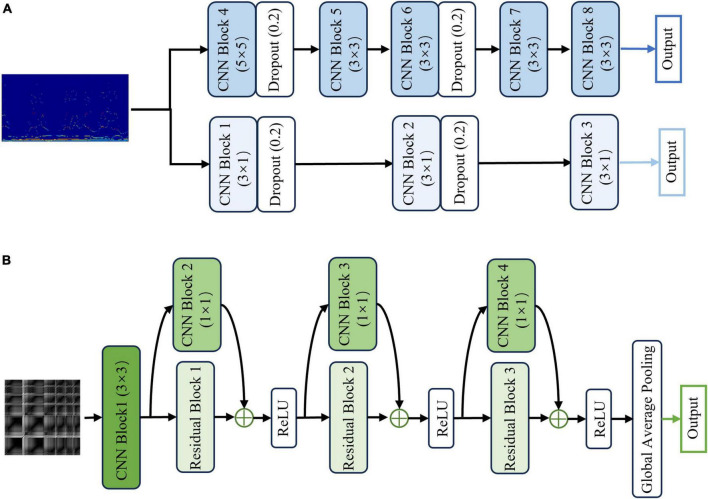
The structure and details of the HS-PCNet module and GRP-ResNet module. **(A)** The overall structure of the HS-PCNet module. **(B)** The overall structure of the GRP-ResNet module.

The first HS-PCNet module inputs the HS built in section 2.2.1 into a parallel two-channel CNN network containing different convolutional kernels. CNN overcomes the limitation of insufficient feature extraction ability of machine learning methods through simultaneous shift calculation of convolutional kernel in the time and frequency dimensions of feature maps (Zhang et al., 2015). It has been used in time-frequency feature extraction of EEG signals (Sui et al., 2021). Therefore, in this section, we use CNN to further extract the high-level time-frequency features of HS. For HS, we design a parallel two-channel CNN network containing different types of convolutional kernels for feature extraction. Two types of convolution kernels are set as [*N*_*kernel*_, 1] and [*N*_*kernel*_, *N*_*kernel*_]. As EEG signals comprise time-series data, we construct a convolution kernel of size [*N*_*kernel*_, 1] to make feature extraction pay more attention to changes in the time domain. The convolution kernel of size [*N*_*kernel*_, *N*_*kernel*_] slides synchronously in the time domain and frequency domain dimensions of the HS to retain its original time-frequency characteristics. The time-frequency features are fully extracted by complementing the high-dimensional features of the two branches. The structure and details of the HS-PCNet module are shown in Figure 6A. The structure and parameter settings in each CNN block of the HS-PCNet module are shown in Table 2. In Table 2, the serial number corresponds to the serial number in Figure 6A. Each CNN block has a batch normalization layer and a ReLU activation layer between the 2D convolution (Conv 2D) and max pooling layers, which are omitted to save space. For the HS-PCNet module, the batch normalization layer normalizes the input data in small batches to speed up the training of the HS-PCNet and reduce the sensitivity to the network initialization. The max pooling layer performs downsampling by dividing the feature map into rectangular pooling regions and calculating the maximum value for each region, which helps reduce overfitting. The dropout layer makes the activation value of a certain neuron stop working with a certain probability, helping to prevent the HS-PCNet from overfitting (Krizhevsky et al., 2017).

**TABLE 2:** The structure and parameter settings in each CNN block of HS-PCNet module.

Index	CNN block
1	Conv 2D: Size (3 × 1), Stride (1 × 1), Filters (8)
	Max Pooling: Size (3 × 1), Stride (2 × 2)
2	Conv 2D: Size (3 × 1), Stride (2 × 1), Filters (16)
	Max pooling: Size (3 × 1), Stride (2 × 1)
3	Conv 2D: Size (3 × 1), Stride (2 × 1), Filters (8)
	Max pooling: Size (3 × 1), Stride (2 × 2)
4	Conv 2D: Size (5 × 5), Stride (2 × 2), Filters (16)
	Max Pooling: Size (5 × 5), Stride (1 × 1)
5	Conv 2D: Size (3 × 3), Stride (1 × 1), Filters (32)
	Max pooling: Size (1 × 1), Stride (1 × 1)
6	Conv 2D: Size (3 × 3), Stride (1 × 1), Filters (64)
	Max pooling: Size (3 × 3), Stride (2 × 2)
7	Conv 2D: Size (3 × 3), Stride (1 × 1), Filters (32)
	Max pooling: Size (2 × 2), Stride (2 × 2)
8	Conv 2D: Size (3 × 3), Stride (1 × 1), Filters (16)
	Max pooling: Size (2 × 2), Stride (2 × 2)

A large number of studies have proved the advantage of residual networks in the field of image recognition (He et al., 2015). Therefore, the second GRP-ResNet module inputs the GRP in section 2.2.2 into a CNN with residual connections to fully learn the nonlinear dynamic features in the GRP. The convolutional module in the GRP-ResNet can use the receptive field of neurons to extract high-level local feature representation of the GRP, and the residual module allows cross-layer propagation, which can avoid overfitting caused by too many layers in the network, and will not lose important information in the feature (He et al., 2015). The overall structure of the GRP-ResNet module is shown in Figure 6B. The structure and parameter settings in each residual block and CNN block are shown in Table 3, and the serial number corresponds to the serial number in Figure 6B. In each block, there is a batch normalization layer after the 2D convolution (Conv 2D) layers, which is omitted to save space.

**TABLE 3:** The structure and parameter settings in each residual block of GRP-ResNet module.

Name	Residual block 1	Residual block 2	Residual block 3	CNN block 1
Details	Conv 2D: Size (3 × 3), Stride (1 × 1), Filters (32)	Conv 2D: Size (3 × 3), Stride (2 × 2), Filters (64)	Conv 2D: Size (3 × 3), Stride (2 × 2), Filters (128)	Conv 2D: Size (3 × 3), Stride (2 × 2), Filters (16)
	ReLU	ReLU	ReLU	ReLU
	Conv 2D: Size (3 × 3), Stride (1 × 1), Filters (32)	Conv 2D: Size (3 × 3), Stride (1 × 1), Filters (64)	Conv 2D: Size (3 × 3), Stride (1 × 1), Filters (128)	Max pooling size (3 × 3), Stride (2 × 2)
Name	CNN block 2	CNN block 3	CNN block 4	–
Details	Conv 2D: Size (1 × 1), Stride (1 × 1), Filters (32)	Conv 2D: Size (1 × 1), Stride (2 × 2), Filters (64)	Conv 2D: Size (1 × 1), Stride (2 × 2), Filters (128)	–

Research shows that the self-attention mechanism (Vaswani et al., 2017) can help the network select important features and assign higher weights to these important features to improve the performance of downstream tasks (Lin et al., 2022; Yang Q. et al., 2023). Therefore, in the third MF-SANet, a feature fusion module based on a multi-head self-attention mechanism is proposed to assign optimal weights to different types of features obtained by the HS-PCNet module and GRP-ResNet module to enhance the information extraction capability of HG-SANet further. The feature fusion formulas are calculated as follows. First, the features extracted from the HS-PCNet module and GRP-ResNet module are concatenated and the concatenated features are denoted as ***Feature_initial***. In the self-attention mechanism, there are three kinds of important input queries, keys and values, denoted as ***QUE***, ***KEY***, and ***VAL***, respectively. They are calculated as Equations 9–11 (Lin et al., 2022):


(9) $${\text{QUE}}_{j}=\text{Feature}\,\_\,\text{initial}\times {\text{W}}_{j}^{\mathrm{QUE}}$$

(10) $${\text{KEY}}_{j}=\text{Feature}\,\_\,\text{initial}\times {\text{W}}_{j}^{\mathrm{KEY}}$$

(11) $${\text{VAL}}_{j}=\text{Feature}\,\_\,\text{initial}\times {\text{W}}_{j}^{\mathrm{VAL}}$$

where *j* = 1, 2, …, *N*_*head*_ and *N*_*head*_ is the number of attention heads. ${\text{W}}_{j}^{\mathrm{QUE}}$, ${\text{W}}_{j}^{\mathrm{KEY}}$, and ${\text{W}}_{j}^{\mathrm{VAL}}$are the parameter matrices. Then, the features of the final output are calculated as Equation 12:


(12) $${\text{Feature}}_{\mathrm{final}}=\text{Concat}\,\left({\text{HEAD}}_{1},{\text{HEAD}}_{2},\ldots ,{\text{HEAD}}_{N_{h\,e\,a\,d}}\right)\,{\text{W}}^{o}$$

where ***W^o^*** is a parameter matric and Concat(⋅) is the concatenating operation. The ***HEAD****_*j*_* is calculated as Equation 13


(13) HEADj=softmax⁢(QUEj⁢KEYjTdK⁢E⁢Y)⁢VALj

where *d*_*KEY*_ is the dimension of keys.

In the classification layer based on the full connection layer, the activation function after the full connection layer is the softmax function.

### 2.3 Experiment configurations

#### 2.3.1 Evaluation metrics

In this paper, epileptic EEG recognition is evaluated using precision (P), recall (R), accuracy (Acc), and specificity (SP) (Sriraam and Raghu, 2017; Gao et al., 2018). The sensitivity and recall are calculated using the same formula, so we no longer calculate sensitivity separately. Precision focuses on evaluating the percentage of true positive samples in all predicted positive samples. Recall focuses on the percentage of all positive samples that are successfully predicted to be positive. Accuracy is the proportion of correctly classified samples in total samples. The specificity is the proportion of all negative samples predicted correctly to all actual negative samples. These metrics are calculated as shown in Equations 14–17.


(14) $$P=\frac{N_{T\,P}}{N_{T\,P}+N_{F\,P}}$$

(15) $$R=\frac{N_{T\,P}}{N_{T\,P}+N_{F\,N}}$$

(16) $$A\,c\,c=\frac{N_{T\,P}+N_{T\,N}}{N_{T\,P}+N_{T\,N}+N_{F\,N}+N_{F\,P}}$$

(17) $$S\,P=\frac{N_{T\,N}}{N_{T\,N}+N_{F\,P}}$$

where *N*_*TP*_ is the number of true positive (TP) samples, *N*_*TN*_ is the number of true negative (TN) samples, *N*_*FP*_ is the number of false positive (FP) samples, and *N*_*FN*_ is the number of false negative (FN) samples.

In the decision stage, the HG-SANet gives prediction labels for all short segments of each sample. Finally, based on the prediction labels of short segments, the majority voting method is used to make the final prediction for the category of each test sample.

#### 2.3.2 Model parameter setting

Parameters of the HG-SANet in the training process are set as follows. Adaptive moment estimation (Adam) optimizer is used to train the HG-SANet. The epoch used for training is 30, and the mini-batch size used for each training iteration is 32. The learning rate is 0.001. The cross-entropy loss function is used as the loss function. We reduce the overfitting of the HG-SANet by adding the regularization term of the weight to the loss function. The number of heads in the attention module is set to 2. In the testing process, the testing sample is input into the proposed system trained by the training set as shown in Figure 3 to obtain the final recognition result. The ten-fold cross-validation is used to obtain an unbiased evaluation of classification performance.

## 3 Results and discussion

### 3.1 Analysis of the proposed model

In this part, we designed several ablation experiments to analyze the effects of each module of the model. First, based on clinical applications and experiments conducted by scholars in the Bonn dataset (Ma et al., 2021), we selected three typical detection tasks to analyze our approach. The three typical tasks are: (1) Two-class detection task: distinguish between set EO and set ES, comparing the performance of methods to distinguish between healthy subjects and epileptic patients. (2) Two-class detection task: distinguish between set SOE and set ES, comparing the performance of methods to distinguish between non-epileptic interictal EEG and seizures in epileptic patients. (3) Three-class detection task: distinguish between normal (include set EO and EC), interictal activities (include set SOE and set SFE), and epileptic seizures (include set ES). This three-class task can be used not only to find epilepsy patients but also to automatically diagnose their symptoms, which is of great significance.

**FIGURE 7:**
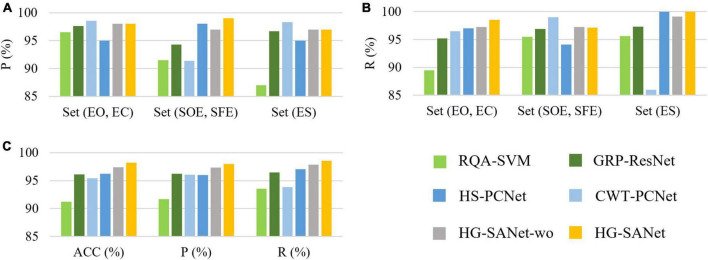
Results of ablation experiments in the three-class detection task of the Bonn EEG time series. **(A)** Precision for each of the three classes. **(B)** Recall for each of the three classes. **(C)** Overall results of the three classes.

In order to verify the performance of each module, we designed the following experiments: (1) Use the RQA method to extract the structural features of RP (Pham, 2020) and input these features into a SVM to classify three-class detection task (denotes as RQA-SVM). (2) A fully connected classification layer is added to the back of the GRP-ResNet module to classify the three-class detection task (denoted as GRP-ResNet). (3) A fully connected classification layer is added to the back of the HS-PCNet module to classify the three-class detection task (denoted as HS-PCNet). (4) The Hilbert Spectrum of the HS-PCNet module is replaced with a CWT-based scalogram (denoted as CWT-PCNet). Then, a fully connected classification layer is added to the back of the CWT-PCNet module to classify the three-class detection task. The Morlet wavelet is used as the mother wavelet (Varlı and Yılmaz, 2023). CWT is an important method for EEG signal analysis. We designed the fourth experiment to compare AOMEMD method and CWT method. (5) The features extracted from the HS-PCNet module and GRP-ResNet module are concatenated and the concatenated features are input to a fully connected classification layer to classify three-class detection task, denotes as HG-SANet without self-attention mechanism (HG-SANet-wo). (6) Use the HG-SANet to classify all three typical tasks. The classification results are shown in Table 4 and Figure 7.

**TABLE 4:** Classification results of the proposed HG-SANet for the three typical tasks.

Cases	Class	P (%)	R (%)	Acc (%)	Mean P (%)	Mean R (%)
Set EO vs. Set ES	Set EO	100	100	100	100	100
	Set ES	100	100			
Set SOE vs. Set ES	Set SOE	99	100	99.50	99.50	99.55
	Set ES	100	99.09			
Set (EO, EC) vs. Set (SOE, SFE) vs. Set ES	Set (EO, EC)	98	98.54	98.20	98	98.56
	Set (SOE, SFE)	99	97.15			
	Set ES	97	100			

Figure 7 shows the results of the ablation experiments designed in this section for the three-class detection task. The average results of the ten-fold cross-validation method are shown in Figure 7. As shown in Figure 7, each module can detect seizures, and the HG-SANet gives the best results in terms of overall performance. The best result of all the 10-fold cross-validation results is 100%. Combining the nonlinear features based on GRP-ResNet with the time-frequency features based on HS-PCNet improves the average accuracy, precision, and recall of the model. Moreover, the average accuracy, precision, and recall of the fusion model with added attention mechanism are increased by 0.8%, 0.67%, and 0.7%, respectively, compared with the fusion model without added attention mechanism. The results in Figure 7 demonstrate the validity of the proposed HG-SANet. The performance of RQA-SVM is the worst. The dimension of the RQA features is only eight. The information expression ability of RQA features is limited. The performance of CWT-PCNet is worse than HS-PCNet. For set ES, the recall of CWT-PCNet is the worst, only 86%. In Table 4, we compare the average performance of the proposed HG-SANet under different classification tasks. As shown in Table 4, in the two-class detection task of identifying set EO and set ES, our method achieves 100% recognition rate.

### 3.2 Comparison with SOTA methods for the classification of epileptic EEG signals

To further validate the effectiveness of the proposed method, we compare the proposed HG-SANet with other state-of-the-art (SOTA) methods on the Bonn EEG time series and the Bern-Barcelona EEG database. The results of the Bonn EEG time series are shown in Table 5. All the comparison methods include deep learning methods and traditional machine learning methods. The results of the proposed HG-SANet in Table 5 are the mean of the 10-cross validation results. As shown in Table 5, the proposed HG-SANet performs best on all the tasks. The proposed HG-SANet has a high recall value, which indicates that the method proposed in this paper can detect the seizure signal as much as possible, which is of great significance for diagnosing the disease. The proposed model can distinguish not only the EEG data of epileptic patients and non-epileptic persons but also the EEG data from epileptic seizures and seizure-free intervals in epileptic patients. When conducting comparative experiments, it was also found that deep learning-based methods outperformed other types of methods.

**TABLE 5:** Comparison of different methods on the Bonn EEG time series database.

Case	References	Methods	Acc (%)	P (%)	R (%)	SP (%)
Set SOE vs. Set ES	Zhao et al. (2020)	Raw EEG + CNN	98.02	–	–	–
	Zeng et al. (2019)	Entropy of visibility heights of hierarchical neighbors +LS-SVM	98.5	–	–	–
	Türk and Özerdem (2019)	CNN + Scalogram	98.5	–	98.01	98.98
	Peng et al. (2021)	Dictionary learning with homotopy	99	–	98	100
	**Proposed**	**HG-SANet**	**99.50**	**99.50**	**99.55**	**99.50**
Set EO vs. Set ES	Jang and Lee (2020)	Wavelet transform+ PSR+ neural network with weighted fuzzy membership	97.5	–	95	100
	Varlı and Yılmaz (2023)	2D CNN + CWT + LSTM	98.97	98.98	98.97	98.97
	Fu et al. (2015)	HHT+SVM	99.13	–	**–**	**–**
	Türk and Özerdem (2019)	CNN + Scalogram	99.5	–	99.0	100
	Zhao et al. (2020)	Raw EEG + CNN	99.52	–	–	–
	**Proposed**	**HG-SANet**	**100**	**100**	**100**	**100**
Set (EO, EC) vs. Set (SOE, SFE) vs. Set ES	Ullah et al. (2018)	Pyramidal one-dimensional CNN	96.27	97.00	95.00	98.00
	Khan et al. (2021)	Hilbert vibration decomposition +LSTM	96.00	95.77	95	–
	Zhao et al. (2020)	Raw EEG + CNN	96.97	–	–	–
	Varlı and Yılmaz (2023)	2D CNN + CWT + LSTM	97.3	97.31	97.30	98.35
	**Proposed**	**HG-SANet**	**98.20**	**98**	**98.56**	**98.55**

The results of the Bern-Barcelona EEG database are shown in Table 6. A binary classification task is performed on this database (focal vs. non-focal). The comparison methods include deep learning, traditional machine learning, and statistical modeling methods. The results of the proposed HG-SANet in Table 6 are the mean of the 10-cross validation results. As seen from Table 6, the performance of the proposed method in epileptic focal location is better than that of all the compared methods. It is also seen on the Bern-Barcelona EEG database that deep learning methods outperform other methods. The results of the two datasets show that the proposed method can classify multiple brain states associated with epilepsy. The proposed method can be used in automatic epileptic seizure detection, the epileptic focal location, and other related applications in diagnosing epilepsy diseases.

**TABLE 6:** Comparison of different methods on the Bern-Barcelona EEG database.

References	Methods	Acc (%)	P (%)	R (%)
Sharma et al. (2015)	Entropy +EMD + SVM	87.00	87.20	90.00
Fasil and Rajesh (2019)	Exponential energy features + SVM	89.00	–	–
Sriraam and Raghu (2017)	Multi-features + SVM	92.15	89.21	94.56
Gao et al. (2018)	Joint time-domain features + auto-regressive linear model + Randomized Power Martingale	–	93.75	93.75
Chen et al. (2019)	STFT + Bhattacharyya distance	–	88.68	94.00
Zhao et al. (2021)	Multi-feature Fusion + FCNN	93.44	94.28	92.50
Sui et al. (2021)	Time-Frequency Hybrid Network	94.30	94.30	94.30
Yang Y. et al. (2023)	Multi-level temporal-spectral features + FCNN	94.50	94.20	95.00
**Proposed**	**HG-SANet**	**95.60**	**95.61**	**95.60**

## 4 Conclusion

In this study, a novel model named HG-SANet is developed for the automated detection of epileptic EEG signals. This innovative model proposes a multi-channel parallel feature extraction module based on multi-domain features and a feature fusion module based on an attention mechanism. Through many experiments, the proposed network structure can capture the non-stationary nonlinear properties of epilepsy EEG well and realize the automatic and high-accuracy detection of epileptic seizures, epileptic focus localization, and EEG classification. The method proposed in this paper is of great significance to detecting and warning brain disease. In the future, we will research other epilepsy-related issues, such as seizure prediction, and further reduce the time complexity of the method and make the method better applied to real-time seizure prediction.

## Data availability statement

The original contributions presented in the study are included in the article/supplementary material, further inquiries can be directed to the corresponding author.

## Ethics statement

Ethical approval was not required for the study involving humans in accordance with the local legislation and institutional requirements. Written informed consent to participate in this study was not required from the participants or the participants’ legal guardians/next of kin in accordance with the national legislation and the institutional requirements.

## Author contributions

CS: Methodology, Writing – original draft, Writing – review & editing, Conceptualization, Data curation, Formal analysis, Validation, Visualization. CX: Writing – review & editing, Methodology, Formal analysis. HoL: Data curation, Writing – review & editing, Formal analysis. HB: Validation, Writing – review & editing. LM: Supervision, Writing – review & editing, Conceptualization. HaL: Supervision, Writing – review & editing, Conceptualization, Funding acquisition, Resources.
